# Therapeutic Effects of the Addition of Fibroblast Growth Factor-2 to Biodegradable Gelatin/Magnesium-Doped Calcium Silicate Hybrid 3D-Printed Scaffold with Enhanced Osteogenic Capabilities for Critical Bone Defect Restoration

**DOI:** 10.3390/biomedicines9070712

**Published:** 2021-06-23

**Authors:** Wei-Yun Lai, Yen-Jen Chen, Alvin Kai-Xing Lee, Yen-Hong Lin, Yu-Wei Liu, Ming-You Shie

**Affiliations:** 1 School of Dentistry, Chung Shan Medical University, Taichung 406040, Taiwan; joe1112joe@hotmail.com; 2 School of Medicine, China Medical University, Taichung 406040, Taiwan; yenjenc.tw@yahoo.com.tw (Y.-J.C.); Leekaixingalvin@gmail.com (A.K.-X.L.); 3 Department of Orthopedics, China Medical University Hospital, Taichung 404332, Taiwan; 4 x-Dimension Center for Medical Research and Translation, China Medical University Hospital, Taichung 40447, Taiwan; roger.lin0204@gmail.com (Y.-H.L.); a0934077552@gmail.com (Y.-W.L.); 5 The Ph.D. Program for Medical Engineering and Rehabilitation Science, China Medical University, Taichung 406040, Taiwan; 6 School of Dentistry, China Medical University, Taichung 40447, Taiwan; 7 Department of Bioinformatics and Medical Engineering, Asia University, Taichung 41354, Taiwan

**Keywords:** fibroblast growth factor-2, magnesium-doped calcium silicate, gelatin, 3D printing, osteogenesis

## Abstract

Worldwide, the number of bone fractures due to traumatic and accidental injuries is increasing exponentially. In fact, repairing critical large bone defects remains challenging due to a high risk of delayed union or even nonunion. Among the many bioceramics available for clinical use, calcium silicate-based (CS) bioceramics have gained popularity due to their good bioactivity and ability to stimulate cell behavior. In order to improve the shortcomings of 3D-printed ceramic scaffolds, which do not easily carry growth factors and do not provide good tissue regeneration effects, the aim of this study was to use a gelatin-coated 3D-printed magnesium-doped calcium silicate (MgCS) scaffold with genipin cross-linking for regulating degradation, improving mechanical properties, and enhancing osteogenesis behavior. In addition, we consider the effects of fibroblast growth factor-2 (FGF-2) loaded into an MgCS scaffold with and without gelatin coating. Furthermore, we cultured the human Wharton jelly-derived mesenchymal stem cells (WJMSC) on the scaffolds and observed the biocompatibility, alkaline phosphatase activity, and osteogenic-related markers. Finally, the in vivo performance was assessed using micro-CT and histological data that revealed that the hybrid bioscaffolds were able to further achieve more effective bone tissue regeneration than has been the case in the past. The above results demonstrated that this type of processing had great potential for future clinical applications and studies and can be used as a potential alternative for future bone tissue engineering research, as well as having good potential for clinical applications.

## 1. Introduction

Functional tissue engineering scaffolds have been under intensive research in recent decades. Researchers have attempted to fabricate biomimetic scaffolds for tissue regeneration in order to resolve clinical difficulties in procedures such as orthopedic surgeries [1]. Massive bone defects have remained a dilemma for surgeons due to the limited self-regeneration ability of bone tissue, which could result in malunion or nonunion of bone [2]. The current gold standard treatment is an autologous bone graft due to its histocompatibility and non-immunogenic properties, which avoid the bone graft suffering from immune rejection [3]. However, the limited availability and invasive harvesting procedure has decreased its applicability. An alternative solution is an allograft, but the risk of immune rejection and disease transmission must be taken into consideration. Therefore, to meet this clinical need, it is crucial to fabricate biocompatible and biodegradable scaffolds with sufficient mechanical properties for bone tissue regeneration [4].

Several materials have been studied for bone tissue engineering, and among these, ceramics have emerged as outstanding bone substitute materials [5]. Nevertheless, numerous studies have gradually focused on the development of ceramic-polymer composites in order to attempt to fabricate different kinds of substitutes for bone tissue engineering [6]. Our previous research reported that gelatin-containing calcium silicate composites have better in vitro osteogenic effects, bioactivity, and degradation rates compared to those of bio-ceramic scaffolds. Calcium silicate is a promising material as a bone substitute because of its obvious biological properties [7,8,9]. According to our previous study, the release of therapeutic ions, SiO32- and Ca^2+^, may induce bone mineralization and regeneration [10]. In addition, an in vitro study showed that calcium silicate-based cements exhibit high apatite-forming abilities and low degradability that are important in bone graft substitutes [11,12]. However, the degradation rate of calcium silicate impedes its application as a tissue-engineered scaffold since it is crucial for scaffolds to be quickly replaced by bone tissue [13]. To solve this problem, the addition of magnesium (Mg^2+^) ion in calcium silicate has made it possible to develop a better biodegradable material. Mg^2+^ is one of the most crucial elements in the human body. It plays an important role in natural bone growth, maintenance, and repair by stimulating osteoblast proliferation [14]. In addition, Mg^2+^ is known for its ability to enable complete bone regeneration, where an in vivo study demonstrated that Mg^2+^ degrades without toxicity in the body and increases osteoconductivity [15]. Furthermore, our previous study indicated that Mg^2+^ content in CS-based materials has a significant effect on the degradability of cements, where the degradation of cements may be controlled by modifications to the Mg^2+^ content [16]. Thus, Mg^2+^-contained CSC was applied in this study to develop an improved biodegradable scaffold. 

The emergence of 3D printing techniques has led to versatile scaffold designs with customized shapes, pore sizes, and morphology. Fused deposit modeling is one of the most popular methods for scaffold fabrication [17,18,19,20]. However, the polymer we applied in fused deposit modeling exhibited hydrophobic properties, which limited cell attachment and proliferation [21]. In order to enhance its biological properties, we decided to simulate the structure of the extracellular matrix (ECM) by surface modifying it with gelatin. Gelatin is a natural compound that is derived from collagen, which is a major component of the ECM [22]. Due to its high levels of biocompatibility, biodegradability, and non-toxicity, gelatin has been widely used in biomaterials, including bone graft materials and as a drug carrier for controlled delivery [23]. Additionally, Chen et al. reported that the presence of gelatin improved the mechanical properties and anti-washout ability of calcium phosphate cement [24]. To achieve better osteogenesis, we further encapsulated fibroblast growth factor-2 (FGF-2) into a gelatin coating, hoping to enhance cell proliferation and differentiation [9]. FGF-2 is a versatile biomolecule that can regulate in various physiological behaviors and supports biological cues to modulate cell adhesion, proliferation, ECM remodeling, and differentiation [25]. 

The purpose of this study is to fabricate porous magnesium-contained calcium silicate scaffolds using a 3D printing technique. The scaffolds were soaked in different concentrations of gelatin solution and formed into a coating membrane, and we further encapsulated FGF-2 into the membrane. The goal was to design novel FGF-2-loaded, Mg^2+^-doped CS scaffolds (MgCS) and to evaluate their material composition and mechanical properties. In addition, the growth-factor-releasing profile and cellular activity of the scaffold are investigated in this study. Furthermore, we cultured the WJMSC on the scaffolds and observe the biocompatibility, alkaline phosphatase activity, and osteogenic-related markers. Finally, in vivo performance was assessed using micro-CT and histological data, which revealed that the hybrid bioscaffolds were able to achieve more effective bone tissue regeneration than their predecessors.

## 2. Materials and Methods

### 2.1. Preparation of the Mg^2+^-Doped Calcium Silicate Powder

The Mg^2+^-doped calcium silicate (MgCS) powders were prepared individually as described previously [16]. Firstly, calcium oxide (CaO, Sigma-Aldrich, St. Louis, MO, USA), silicon dioxide (SiO2, Sigma-Aldrich, St. Louis, MO, USA), and magnesium oxide (MgO, Sigma-Aldrich, St. Louis, MO, USA) powders were weighed separately and placed into 100% alcohol, after which, the mixtures were then ball milled for 1 h at 300 rpm and placed into a 100 °C oven to dehydrate the alcohol. The mixtures were then placed in a sintering furnace at a temperature of 1400 °C for 2 h, where the temperature was raised in increments of 10 °C per minute for the purpose of sintering, after which the temperature was then naturally cooled to room temperature.

### 2.2. Ceramic Ink Preparation and Manufacturing of MgCS Scaffolds

The MgCS ceramic powders were added into 100% alcohol according to the required proportions and stirred for 24 h, after which polycaprolactone (PCL, molecular weight = 43,000, Polyscienece, Warrington, PA, USA) was added. The mixture was heated to 100 °C to allow complete evaporation of the alcohol. The ratio of PCL to the ceramic powders was 50:50. Prior to printing, the pastes were loaded into syringes with a 400 μm needle, and our in-house GeSiM 3D bioprinter (BioScaffolder 3.1, GeSiM, Grosserk-mannsdorf, Germany) was used to design the porous scaffolds, then generate the g-code of printing path and upload to the machine. Take the scaffold for the compression test sample as an example. Each layer had seven struts, where the layers were stacked perpendicular to every two layers, and each strut or pores was designed to be 500 μm apart with a strut height of 300 μm. The printing temperature was set at 80 °C; the compression and expansion pressure were set at 400 kPa per second, and the printing rate was 1 mm/s in order to develop uniform, porous scaffolds. We allowed the material to cool down for 30 s after printing each layer before continuing. According to the setting of the machine, we finally collected 48 samples at a time from the printing platform. It took about 30 min to print each sample.

### 2.3. Preparation of the Gelatin-coated Scaffold and Genipin Solution

The gelatin used in this study was porcine type A gelatin (Sigma-Aldrich, St. Louis, MO, USA) that was dissolved in 60 ℃ deionized water (2.5 wt% and 5.0 wt%) and stirred until it was completely dissolved. The prepared gelatin-coated scaffolds were soaked in the gelatin solution and left for 5 min to allow complete penetration of the gelatin into the scaffolds. The scaffolds were then removed from the gelatin solution and left to dry for 5 min. The groups were labeled as G0, G2.5, and G5.0 to represent coating with 0, 2.5 and 5.0% gelatin solution, respectively. The gelatin-coated 3D scaffold was immersed in the 0.02 wt% genipin solution (Challenge Bio, Yun-Lin, Taiwan) to stimulate cross-linking reactions for 24 h. It was then removed and washed with deionized water to rinse off the excess genipin, after which the scaffolds were placed in a freeze dryer for 24 h before subsequent assays.

### 2.4. Characterization of the Scaffolds

The scaffolds were placed onto a microscope slide, and a droplet of 5 μL of deionized water was pipetted on the scaffolds. A desktop microscope was then used to take photo images of the water droplets, which were analyzed using the contact angle protractor from the computer image analysis software (Image J). High-resolution X-ray diffractometry (XRD; Bruker D8 SSS, Karlsruhe, Germany) at a scan speed of 1° per minute was also used to detect the crystalline phase composition of the scaffold surface in the 2θ range from 20° to 60°. A Fourier-transform infrared spectrometer (FTIR, Bruker, Germany) was used to assess the functional groups of the scaffolds from 500 cm^−1^ to 4000 cm^−1^, and a prime Schottky field emission scanning electron microscope (SEM, JEOL, Tokyo, Japan) was used to observe the microscopic morphology of the scaffolds. The mechanical experiment was performed using an EZ-Test machine (Shimadzu, Kyoto, Japan). During the experiment, compression was performed at a speed of 2 mm/min. The Young’s modulus and compressive strength of the scaffold were calculated from the stress–strain curve. Six independent sets of the test were carried out and the average was recorded.

### 2.5. Degradation Appraisement of the Fabricated Scaffolds

The in vitro degradation test was carried out by observing the weight loss of the scaffolds after immersion in 37 °C 50 mL of a simulated body fluid (SBF) for 3 months. The ionic composition of the SBF solution was similar to that of human plasma, consisting of 7.9949 g of NaCl, 0.2235 g of KCl, 0.147 g of K_2_HPO_4_, 0.3528 g of NaHCO_3_, 0.071 g of Na_2_SO_4_, and 0.2775 g of CaCl_1_, and 0.305 g of MgCl_2_ dissolved in 1000 mL of distilled water adjusted to pH 7.4 with hydrochloric acid and tris(hydroxymethyl)aminomethane. The scaffolds from each group were immersed in SBF in 50 mL centrifuge tubes. The original weight of the scaffolds was measured on a weighing scale before immersion in 75 wt% alcohol for 30 min, after which six scaffolds from each group were rinsed with deionized water and placed into the SBF solution in a centrifuge tube. The scaffolds were removed from the SBF after 1, 2, and 3 months of immersion, gently washed, dried in an oven for 24 h, and weighed. In addition, the FE-SEM was used to observe hydroxyapatite formation on the surfaces of the scaffolds.

### 2.6. Cell Viability Assay and Fluorescent Staining

Wharton Jelly Mesenchymal stem cells (WJMSC; ScienCell Research Laboratories, Carlsbad, CA, USA) used in this study were cultured in a commercial medium (#7501, ScienCell Research Laboratories, Carlsbad, CA, USA) until passages 3–8. The cells were cultured on the scaffold at a cell concentration of 10^6^ cells per mL and placed in an incubator in a 37 °C humidified atmosphere and 5% CO2. The medium was changed every 2–3 days and cell viability was assessed using PrestoBlue (Invitrogen, Carlsbad, CA, USA). Briefly, 30 μL of PrestoBlue solution: 270 μL of medium was added to the scaffold and incubated in a 37 °C incubator for 30 min. A total of 100 μL of the mixture solution was then transferred from the culture plate to a 96-well microplate. A spectrophotometer (Infinite Pro M200, Tecan, Männedorf, Switzerland) was used to read the absorbance at a wavelength of 570 nm with a reference wavelength of 600 nm. 

After 1 and 7 days of culture, fluorescence staining was done to observe the cellular morphology. Firstly, the medium was removed, and the scaffolds were gently rinsed with phosphate buffered saline (PBS, Invitrogen, Carlsbad, CA, USA) to remove any excess culture medium. The cells were then fixed with 4% paraformaldehyde for 30 min and rinsed with PBS solution to remove any excess paraformaldehyde. A total of 0.1% Triton X-100 (Invitrogen, Carlsbad, CA, USA) in PBS was then added for 10 min to allow subsequent dye permeation, and 1:500 phalloidin (Alexa Fluor 488, Invitrogen, Carlsbad, CA, USA) was added. The mixture was then shaken for 1 h in the dark, the scaffolds were gently rinsed with PBS solution to remove excess solution, and DAPI fluorescent dye was added and left to react for 20 min in the dark. A confocal microscope (Leica TCS SP8, Wetzlar, Germany) was utilized to observe the cells in the dark state.

### 2.7. Osteogenesis Capabilities

In the osteogenic assay, the WJMSC were cultured with osteogenesis assay kits (StemPro™ osteogenesis differentiation kit, Invitrogen, Carlsbad, CA, USA) to estimate cell differentiation. For the measurement of early osteogenic differentiation, alkaline phosphatase activity was tested after 3 and 7 days. Briefly, cells were immersed in 50 μL of 1% NP40 Cell Lysis Buffer to lyse the cells, and the pNPP alkaline phosphatase assay kit (BioAssay Systems, Biocore, NSW, Australia) was used for determination. Total protein content was measured using the BCA protein detection kit (Thermo Scientific, Waltham, MA, USA). Afterwards, the relative alkaline phosphatase activity was calculated as the change in absorbance divided by the total protein content. In addition, according to the manufacturer’s instructions, bone sialoprotein (BSP) and osteocalcin (OC) enzyme-linked immunosorbent assay kits (Invitrogen, Carlsbad, CA, USA) were used to quantify the BSP, and OC proteins expression of WJMSC were cultured on different scaffolds for 7 days and 14 days, respectively. All of the cultivation runs were performed three times.

### 2.8. FGF-2 Loading, Release Kinetics, and Influences on Cell Behavior

Basic fibroblast growth factor, also known as FGF-2, was loaded onto the scaffolds and the release profiles of FGF-2 were evaluated to determine the release kinetics of the scaffolds. FGF-2 (ProSpec-Tany TechnoGene, Rehovot, Israel) was mixed with distilled water to achieve a concentration of 500 ng/mL. The various scaffolds were then immersed in the FGF-2 solution for 2 h, for loading of FGF-2. After which, the scaffolds were immersed in medium at 37 °C for various durations to evaluate release profiles of FGF-2 using an enzyme-linked immunosorbent assay (ELISA) kit (Invitrogen, Carlsbad, CA, USA). The concentration was quantified using absorbances measured using a spectrophotometer. Finally, influence of the FGF-2-loaded scaffolds on cell proliferation and osteogenesis was also evaluated.

### 2.9. Establishment of a Critical-Sized Bone Defect Model

The animals used in this study were New Zealand male white rabbits with an approximately weight of 1.8 kg. They were fed and housed according to the protocol established by the Animal Experiment Center of China Medical University (CMUIACUC-2018-090). In the experimental procedure, the rabbits were first anesthetized, and then a gas anesthesia machine was used to continuously anesthetize the rabbits with 5% isoflurane/oxygen to prevent them from waking up during the surgery. After shaving the hair and disinfecting the rabbit’s hind legs, the skin from the thigh extending to the inner side of the lower leg and along the muscle was cut with a scalpel to expose the lateral femoral condyle. To create a critical-sized bone defect, a hole with diameter of 6 mm and a depth of 6 mm on the femoral condyle was made using a dental handpiece. Subsequently, after removing broken bones in the surgical site with PBS surgical probes, scaffolds were implanted into the defect site, and then the wound was closed and anti-inflammatory ointment was applied along the suture. The animals were sacrificed in the fourth and eighth weeks after implantation, followed by subsequent analysis.

### 2.10. Micro-Computed Tomography (µ-CT) and Histological Analysis

The µ-CT images of the condyle femur wrapped scaffold were scanned using the multi-scale X-ray nano-CT System (SkyScan 2211, Bruker, Belgium) at a voltage of 100 kVp, a current of 330 μA, and an output of 20 Watts. The images were reconstructed using reconstruction software (Insta Recon, Bruker, Belgium). The software AvizoFire 8.0 (Visualization Sciences Group, Bordeaux, France) was employed to analyze the bone volume (BV/TV) and the trabecular thickness (Tb.Th) of the bone. After the µ-CT scan was completed, the sections were appropriately divided into the appropriate thickness using a frozen section procedure. The sections were stained with hematoxylin-eosin (HE); the collagen fibrils were stained using Masson’s trichrome (MT), and the calcified bone was stained in dark-red using von Kossa (VK). Lastly, the samples were observed with an optical microscope.

### 2.11. Statistical Analyses

A one-way statistical analysis of variance (ANOVA) was applied to analyze the significance of the differences among the various experimental groups in each experiment. Three sets of experimental repeats were done for each test, and the averages were recorded. Determination of a significant deviation in each sample was made using Scheffe’s multiple comparison test. A *p*-value < 0.05 was considered statistically significant, and is indicated by “*”.

## 3. Results and Discussion

### 3.1. Contact Angle

**Figure 1:**
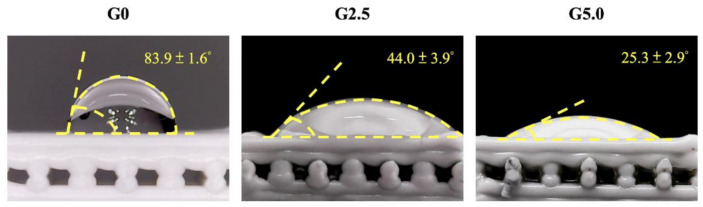
Contact angle of the gelatin-coated MgCS scaffolds.

The water-in-air contact angle on the surfaces of the various scaffolds with and without the gelatin modification are shown in Figure 1. A water droplet was pipetted onto the surfaces of the scaffolds, and the water contact angle indicated the wettability of the biomaterials. This test is a common, easy method to assess the hydrophobicity or hydrophilicity of biomaterials [26]. In general, an angle of more than 90° indicates that the biomaterial is hydrophobic, and vice versa. As seen in Figure 1, G0 had a contact angle of 83.9 ± 1.6°; G2.5 had a contact angle of 44.0 ± 3.9°, and G5.0 had a contact angle of 25.3 ± 2.9°. It can be seen that the contact angle decreased with increases in the gelatin concentration, with G5.0 having approximately a 70% reduction in the contact angle as compared to G0. Gelatin has a hydrophilic amine and carboxyl groups in its protein chains, thus explaining the improvement in hydrophilicity [27]. In a recent study done by Yu et al., it was concluded that cell adhesion and proliferation are significantly influenced by hydrophilicity. However, inhibition of cellular adhesion was noted on the hydrophobic surfaces [28]. In addition, Joo et al. modified the surface of poly(dimethylsiloxane) with hyaluronic acid and gelatin and showed that such a modification further enhanced the hydrophilicity of biomaterials and led to subsequent increased cellular activities and tissue regeneration [29].

### 3.2. Characterization of Chemical Composition of the Scaffolds and Its Bioactivity

**Figure 2:**
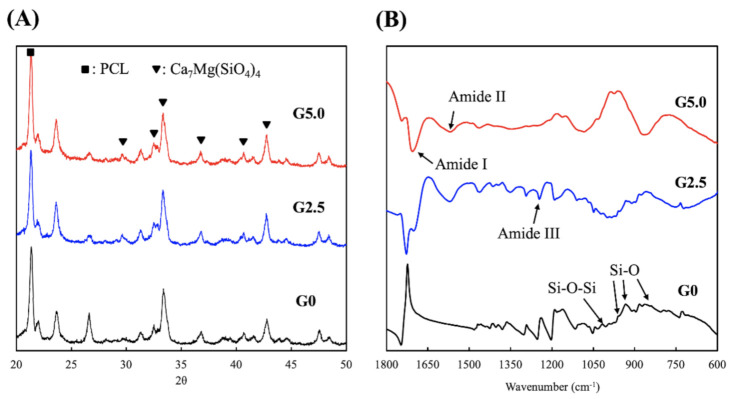
(**A**) X-ray diffractometry (XRD) and (**B**) Fourier-transform infrared spectrometer (FTIR) spectra of the gelatin-coated MgCS scaffold.

The XRD and FTIR analysis for the various scaffolds is shown in Figure 2. As shown in Figure 2A, all three scaffolds had a PCL peak (21.5o) and multiple Ca_2_Mg(SiO_4_)_4_ peaks, as noted by the inverted triangle signs. These XRD signals of PCL and Ca_2_Mg(SiO_4_)_4_ were similar to those reported by other scholars, thus indicating that there was successful incorporation of Mg^2+^ ions into the CS [30]. In addition, surface modification with gelatin did not affect or change the original structural components of MgCS, thus making it possible to retain the advantageous characteristics of MgCS. The MgCS scaffold surfaces were modified with gelatin via a chemical cross-linking method with genipin. The FTIR results from Figure 2B confirmed the successful gelatin conjugation with MgCS, as seen from the presence of amide bonds in G2.5 and G5.0. All spectra contained Si-O and Si-O-Si peaks, which is characteristic of the presence of CS. Amide peaks noted at 1750, 1600, and 1300 cm^−1^ showed that gelatin was successfully incorporated into the MgCS [31].

**Figure 3:**
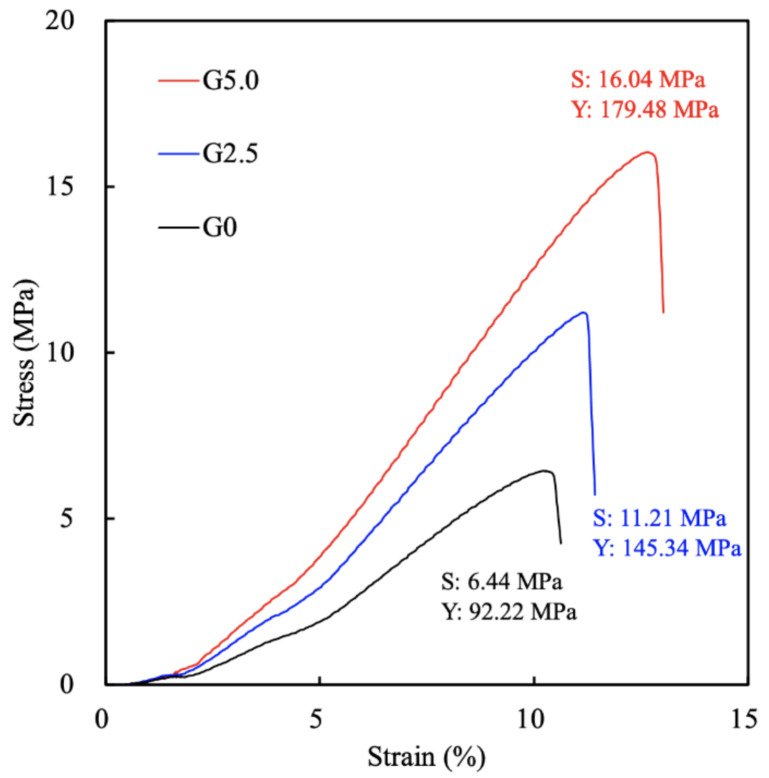
Stress–strain curves of the gelatin-coated MgCS scaffolds.

Figure 3 shows the SEM images of the scaffolds and the struts, where it can be seen that the scaffolds had well-arranged pore structures with completely interconnected pores. In addition, the layers were stacked neatly on top of one another, and there were no collapsed or weak structures. The stability of the printing process and reproducibility are important factors to consider if we were to proceed with animal and human studies. Precision medicine is an emerging aspect of medicine whereby individual variability and personalization is at the core of treatment principles. In addition, the stability of the scaffolds is directly related to mechanical strength and porosity of the scaffolds, of which reports had been made stating that certain cellular activities (cell proliferation and mineralization) were influenced by pore sizes and interconnectivity of pores. For our case, a pore size of 100–400 µm is highly recommended for bone tissue regeneration as pores of this range were reported to bring about enhanced cellular adhesion, attachment, migration and reduced aggregation of cells [32]. A magnified view of the struts made it possible to look for the presence of the gelatin coating on the surfaces of the scaffolds. The surface of G0 was smooth, and G2.5 and G5.0 had roughened surfaces with agglomerations. Surface characteristics play an important role in influencing cellular behavior and activities. Results have shown that bone regeneration is enhanced when the surfaces of scaffolds are coated with silk, calcium phosphate, or hydroxyapatite molecules, and such modifications are known to affect surface roughness [33]. Increased surface roughness is related to increased surface area to which cells can adhere and attach to during early seeding. Wang et al. showed that titanium modification increases surface roughness, thus supporting osteo-integration by enhancing osteoblast differentiation and release of local factors [34]. Therefore, in the present study, it was hypothesized that modification with gelatin would enhance bone regeneration due to greater surface roughness and improved hydrophilicity in the scaffolds.

### 3.3. Effects of Mechanical Strength and Degradation Properties on the Soaking Experiments

**Figure 4:**
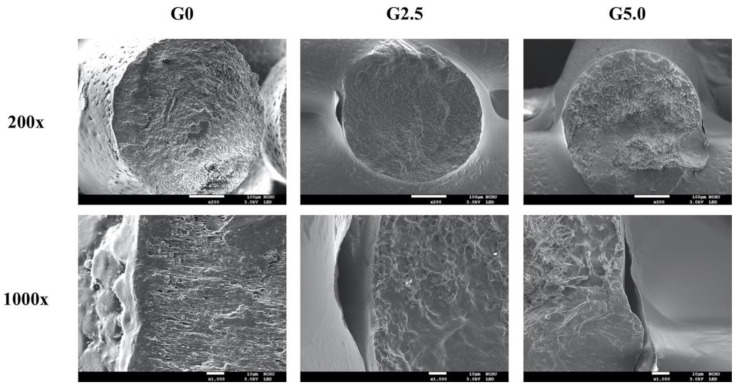
SEM images of the surfaces of the gelatin-coated MgCS scaffolds.

Figure 4 shows the stress–strain curves of the various scaffolds with the Young’s modulus. G0, G2.5, and G5.0 had tensile strengths of 6.44 MPa, 11.21 MPa, and 16.04 MPa, respectively. The addition of gelatin led to an approximate 1.8 to 2.6 times increase in tensile strength. In addition, G0, G2.5, and G5.0 had Young’s moduli of 92.22 MPa, 145.34 MPa, and 179.48 MPa, respectively. It was noted that the mechanical strength of the scaffolds increased exponentially with increases in the gelatin concentration. The improved hydrophilicity did not have any adverse effects on the mechanical strength of the scaffolds; instead, it improved their tensile strength. This result indicated that the appropriate amount of gelatin coating could enhance the mechanical properties of the scaffolds, which was possibly attributable to a better distribution of the mechanical load [24]. Sohrabi et al. proposed that the amide and carboxyl groups in gelatin react with the scaffold, thus leading to increases in tensile strength [35]. The increased mechanical strength allows for better handling of the scaffolds during surgeries and also prevents implant failure due to the native stress on bones.

**Figure 5:**
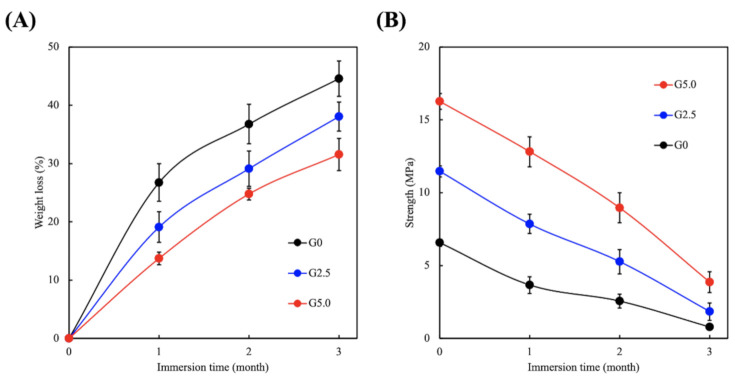
(**A**) Weight loss profiles and (**B**) tensile strength of the gelatin-coated MgCS scaffolds.

The scaffolds were immersed in PBS for 3 days, and biodegradability was assessed by determining weight loss of the scaffolds. The degradation rate and mechanical strength of the scaffolds after immersion are shown in Figure 5. It can be seen that the weight loss was about 26% (1 month), 35% (2 months), and 43% (3 months) for G0 and 12% (1 month), 22% (2 months), and 27% (3 months) for G5.0 (Figure 5A). In terms of mechanical strength, G0 was 4.6 MPa (1 month), 2.6 MPa (2 months), and 0.5 MPa (3 months), and G5.0 was 13.0 MPa (1 month), 10.0 MPa (2 months), and 5.2 MPa (3 months) (Figure 5B). As for the decreased strength after reaching the maximum point, the degradation process may be a factor, as discussed above [24]. These results indicated that the gelatin modification slows down the degradation of MgCS scaffolds, which makes such scaffolds more appropriate for bone regeneration applications. A good scaffold should degrade at the same rate of regeneration as that of the tissue so as to support tissue regeneration. Furthermore, newly regenerated tissues are generally weaker than healthy tissues, so the scaffold should still be able to provide sufficient mechanical support during the early stages of regeneration [36]. With such concepts in mind, we hypothesized that the gelatin-modified MgCS scaffolds had better potential for supporting bone tissue regeneration as compared to neat MgCS scaffolds.

**Figure 6:**
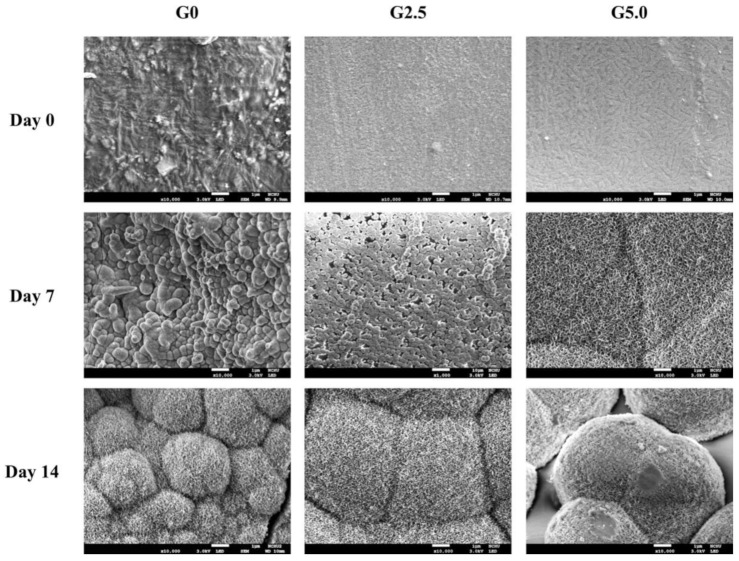
SEM images of hydroxyapatite precipitated on the gelatin-coated MgCS scaffolds after 0, 7, and 14 days of immersion in the culture medium. The white bar below dictates 1 µm.

The development of the hydroxyapatite layer formation on the surfaces of the scaffolds over 14 days of immersion are shown in Figure 6. On day 0, it was noted that all the surfaces were clean or smooth with no aggregation of hydroxyapatite. On day 7 of immersion, the aggregates of hydroxyapatite precipitation were noted in all groups. For the purpose of a better comparison, a higher magnification of 10,000× was used to observe the hydroxyapatite formation on the G0 group instead of the 1000× magnification used for the other groups. After 14 days of immersion, hydroxyapatite aggregations could be observed in all groups. However, G5.0 had larger clusters of aggregations as compared to the other two groups, thus indicating that there was more hydroxyapatite formation in the G5.0 group. In addition, it was noted that the size and thickness of hydroxyapatite formation increased with longer SBF immersion time. Several studies have indicated the calcium ions released from calcium silicate-based materials will react with phosphate ions in SBF and mineralized precipitation on scaffolds to form a hydroxyapatite layer that has been demonstrated to support subsequent bone tissue-implanted scaffold bonding [37]. Bone is a heterogeneous composite material with constituents including hydroxyapatite mineral, a mixed organic component (type I collagen, lipids, and non-collagenous proteins), and water [38]. Scientists have since determined that the essential requirement for a scaffold to bond to living bone is the formation of this hydroxyapatite layer on the surfaces of interest [39]. In addition, this layer is similar to bone mineral in terms of composition and structure; therefore osteoblasts have been shown to prefer such an interface for proliferation and subsequent bone regeneration [40]. It was therefore hypothesized that the presence of additional amide and carboxyl groups from the gelatin contributed to the increased hydroxyapatite formation.

### 3.4. Cell Proliferation of WJMSC Cultured on Scaffolds

**Figure 7:**
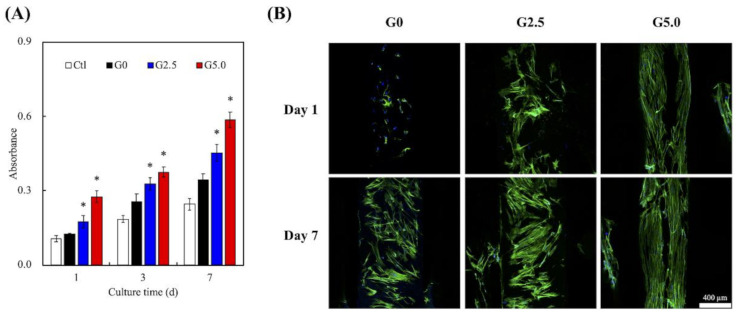
(**A**) Cell proliferation and (**B**) F-actin stain of WJMSC on the gelatin-coated MgCS scaffolds at various time points. The scale bar is 400 µm.

The proliferation rates and F-actin staining of cells cultured on the scaffolds were assessed, as shown in Figure 7. Gelatin has tripeptide arginine-glycine-aspartate (RGD) motifs, which act as receptors for cellular adhesion factors such as integrin. As shown in Figure 7, G0 had a higher proliferation rate as compared to Ctl at all time points, and increasing the concentrations of gelatin led to significantly higher levels of proliferation at all time points. This significance was noted even after 1 day of culture, with G2.5 and G5.0 having 15% and 35% increments of improvement, respectively, as compared to G0. It was hypothesized that increasing the gelatin concentration led to increased RGD motifs, thus leading to enhanced initial cellular adhesion and proliferation. In fact, a MgCS scaffold is already a material that can promote cell growth, mainly because it spontaneously releases Si ions to affect the growth and differentiation of various primary cells [11,41,42]. In addition, the presence of gelatin increased the hydrophilicity and surface roughness, which also had a part to play in up-regulating cellular proliferation. F-actin results in Figure 7B further confirmed that G2.5 and G5.0 had a higher number of cells after 1 and 7 days of culture, as seen from the increased F-actin staining. Furthermore, the cells were spread homogeneously on the surfaces of the scaffolds, and the cells were better adhered onto the surfaces of G2.5 and G5.0 as observed from the F-actin stain. We had previously shown that the surfaces of hydrophobic materials can be positively modified by adding a layer of hydrophilic substrate onto the surfaces. By doing so, we managed to change the critical surface tension values, thus leading to enhanced cellular adhesion and proliferation. In addition, the adhesion and growth of WJMSC in the G5.0 group were better than those in the pure MgCS scaffold (G0), indicating that the gelatin layer provides a biomimetic microenvironment that mimics the natural bone matrix in the 3D-printed MgCS scaffold. Similarly, Martínez-Vázquezet et al. modified the surface of titanium with phosphorylated gelatin, and their results showed that cell adhesion increased with increased concentrations of gelatin [43]. However, our study is the first to incorporate gelatin modification into a 3D-printed MgCS scaffold.

### 3.5. Bone Regeneration-Related Protein Expression

**Figure 8:**
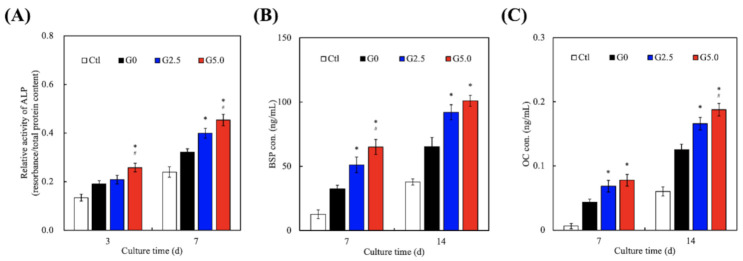
(**A**) ALP; (**B**) BSP; (**C**) OC expression of WJMSC cultured on the gelatin-coated MgCS scaffolds at various time points. * indicates a significant difference (*p* < 0.05) when compared to G0. # indicates a significant difference (*p* < 0.05) when compared to G2.5.

Osteogenesis-specific factors such as alkaline phosphatase (ALP), bone sialoprotein (BSP), and osteocalcin (OC) were assessed, for which the results are shown in Figure 8. It can be observed that G0 again had higher levels of ALP, BSP, and OC at all time points as compared to Ctl. However, G50 had significantly higher levels of ALP, BSP, and OC after 3 and 7 days of culture as compared to G0. G5.0 had 1.3 times (day 3) and 1.8 times (day 7) higher ALP as compared to G0. Similarly, G5.0 had 1.5 times (days 3 and 7) higher BSP and 1.4 times (days 3 and 7) higher OC as compared to G0. The results for G2.5 were approximately in the middle of G0 and G5.0. ALP is known to be involved in matrix mineralization and is specific to osteoblasts. Reports have been made stating that ALP is mainly involved in the post-proliferative phase of osteogenesis, which has been estimated to be approximately days 12–15. OC is also found post-proliferatively at the start of matrix mineralization [44]. In addition, BSP makes up 10% of the non-collagenous protein in the bone matrix and represents late stage osteoblastic differentiation and early stage matrix mineralization [45]. These results confirm the osteogenesis differentiation of WJMSC by the expression of ALP, BSP, and OC, which are considered important osteogenic-related markers of bone cells and further provide a functional demonstration of bone regeneration. Therefore, it could be inferred that G5.0 had more osteo-inductive effects as compared to G2.5 and G0.

### 3.6. Effect of FGF-2 Release from Gelatin-Coated MgCS Scaffolds

**Figure 9:**
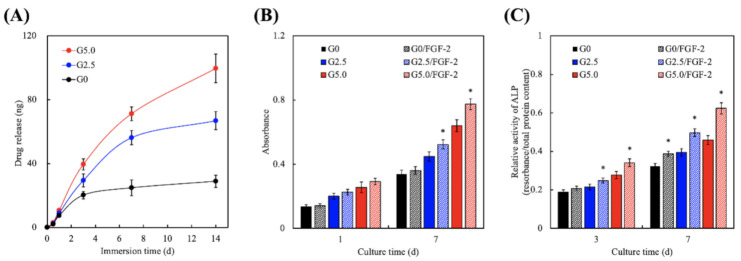
(**A**) Drug release profiles; (**B**) proliferation assay; (**C**) ALP expressions of WJMSC on the gelatin-coated MgCS scaffolds at different time points. * indicates a significant difference (*p* < 0.05) when compared to G0.

The FGF-2 release profiles of the 3D-printed gelatin-coated MgCS scaffolds are shown in Figure 9A. It can be seen that G0 showed a burst release profile of FGF-2 during the first 3 days, followed by a gradual release over the next 14 days. The release rate was clearly reduced in the G0 scaffold, and the release amounts of FGF-2 were approximately 20.2 ± 2.4 ng and 28.9 ± 3.8 ng at incubation periods of 3 and 14 days, respectively. In this group, the FGF-2 released from G0 during the first 3 days was mainly attributed to desorption from the surface of the MgCS scaffold [46]. However, FGF-2 was continuously and stably released to achieve the effect of promoting tissue regeneration in G2.5 and G5 scaffolds. After immersion for 14 days, G2.5 and G5.0 released 66.9 ± 5.7 ng and 99.7 ± 8.9 ng of FGF-2, respectively. The biggest difference among the three groups was that the release profiles of FGF-2 in G2.5 and G5.0 were still rising. It was hypothesized that the presence of gelatin coating allowed for higher amount of FGF-2 loading, thus leading to increased release of FGF-2 upon immersion. Gelatin is a common material used for loading of factors as gelatin is generally non-toxic, inexpensive and the coating and cross-linking processes are considered simple as compared to other materials [47]. The loaded FGF-2 was capable of gradually releasing from G2.5 and G5.0 due to the fact that the MgCS scaffold was buried in the gelatin matrix, which subsequently delayed it from contact with the solution. The results of the cell proliferation assay indicated that the quantification of WJMSC cultured on the scaffolds exhibited a gradual rising trend when more gelatin was coated on the scaffold. However, the printed MgCS scaffold with a thicker gelatin coating and also FGF-2 immersion by cultivation of WJMSC indicated superior cell expansion on all test days. The data in Figure 9B show significant differences in the G2.5/FGF-2 group and the G5.0/FGF-2 group compared with their non-coated groups after 7 days of cultivation. In addition, the in vitro ALP activity of WJMSC cultured on the G0, G2.5, and G5.0 scaffolds without/with FGF-2-loading was accessed using the ALP kit for estimation of early osteogenic differentiation at the tested time points. The examination of the explanted solution measured via a microplate reader revealed that the increasing values were in response to the gelatin concentration. These results were similar to those obtained for the proliferation assay, in which the measurement (Figure 9C) showed significant osteo-differentiation in the G0/FGF-2, G2.5/FGF-2, and G5/FGF-2 groups from days 3 to 7. Such outcomes could be due to the fact that the concentration of coated gelatin determined by the surface properties involves thickness directly influencing adsorption and the gradual release of FGF-2 with different concentrations of gelatin coating [48]. Studies have shown that FGF-2 signaling plays an important role in the FGF receptor (FGFR) of precursor cells, effectively predetermining FGFR expression, the stages of cell maturation, and various microenvironments [41]. The FGF-2 signal promotes the proliferation of precursor osteoblasts, thereby expanding the cell population and increasing the proportion of osteoblasts in the bone environment.

### 3.7. Micro-CT Evaluation in Critical-Size Bone Defects in a Rabbit Model

**Figure 10:**
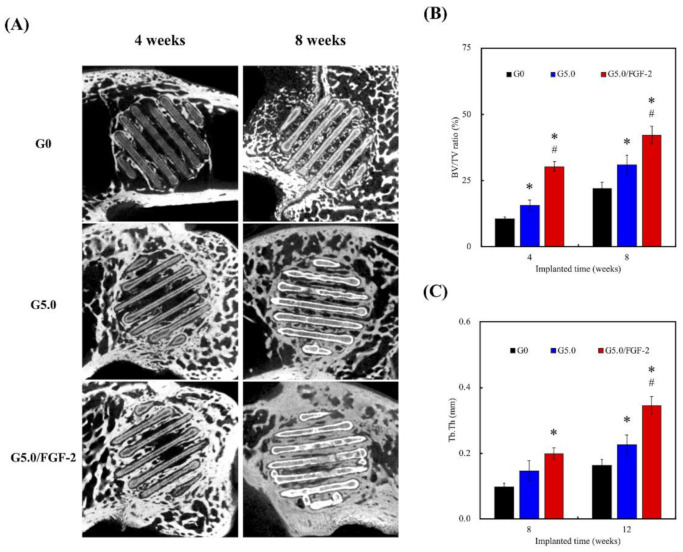
(**A**) Micro-CT image of the bone defects; (**B**) the quantified bone volume/total volume (BV/TV); (**C**) micro-CT quantified trabecular thickness (Tb.Th) analysis at various time points. * indicates a significant difference (*p* < 0.05) when compared to G0. # indicates a significant difference (*p* < 0.05) when compared to G5.0. The scale bar is 2 mm.

The in vivo regenerative capabilities of the gelatin-modified scaffolds were assessed using rabbit femur fracture models. After 4 and 8 weeks of implantation, the tissues around the scaffolds were dissected and analyzed using micro-CT and computer-aided quantification. Micro-CT is commonly used for small animals since it is sufficient to provide a high temporal and spatial resolution imaging of small animals. As shown in Figure 10A, after 4 weeks of implantation, G5.0 had new bone tissues (hyperdense) growing and migrating into the center of the scaffold, and G0 had less hyperdense growth amongst the struts. After 8 weeks, the struts of G5.0 were interrupted and non-contiguous as the new bone tissues began to grow and invade the struts. There was an obvious loss of volume and density in the G5.0 scaffolds. New bone tissue had started growing into the center of the G0 scaffolds; however, the tissues had not yet started to invade the struts, and the new bone tissues were at a lower density than the new bone tissues in G5.0. In addition, the micro-CT images at week 8 further confirmed the degradation results above. The scaffolds were still present even after 8 weeks of implantation, thus stating that the porous scaffolds guided tissue regeneration and provided ample mechanical support for bone regeneration. Similarly, the quantification results showed that G5.0 had significantly higher bone volume and trabecular thickness at all time points as compared to G0. In addition, G5.0 was noted to have significantly enhanced bone volume as compared to G2.5 at both weeks 4 and 8. In terms of trabecular thickness, G5.0 only had significantly increased trabecular thickness after 8 weeks of implantation. Bone volume was used to measure the area that was newly occupied with mineralized bones, and trabecular thickness was used to assess the thickness of newly formed cancellous bone tissues. The overall effects of gelatin modification, such as increased hydrophilicity, increased surface roughness, and the ability to provide natural RGD motifs could be clearly observed in the results discussed above.

### 3.8. Immunohistochemistry in Critical-Size Bone Defect in Rabbit Model

**Figure 11:**
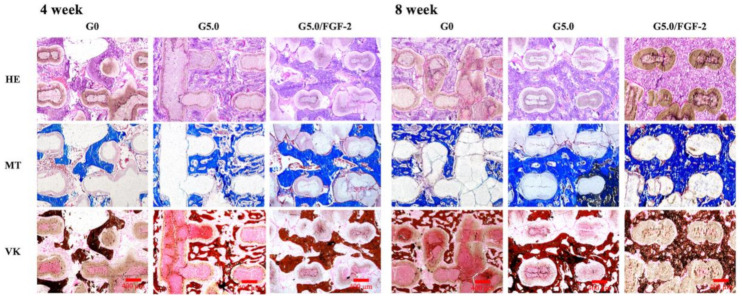
Hematoxylin-eosin (HE); Masson’s trichrome (MT); von Kossa (VK) histological analyses of the gelatin-coated MgCS scaffolds after implant into the bone defects for 4 and 8 weeks. The scale bar is 400 µm.

A histological analysis was done to provide a more detailed analysis of the bone response to the scaffolds with or without gelatin, the results of which are shown in Figure 11. In the HE staining, purple represents newly regenerated tissues, and the intensity represents the number of regenerated tissues. As can be observed, the tissue regeneration in G5.0 was well formed around the struts of the scaffolds on both weeks 4 and 8, whereas there was only minimally regenerated tissue in the G0 group. In addition, the HE staining for G5.0 on week 8 was homogenous and organized, whereas that for G0 was disorganized and scattered. Furthermore, newly regenerated bone tissues had begun to infiltrate the struts of the scaffolds for G5.0 at week 4, but there was little or no infiltration of the struts for G0 even at week 8. In addition, the histologic images of G5.0 and G5.0/FGF-2 scaffolds did not indicate any inflammatory infiltration. During the manufacturing process, the gelatin-coated MgCS scaffolds were treated with genipin, which caused cross-linking of the gelatin. Therefore, for the gelatin-coated MgCS scaffolds in this research, no adverse response was found in terms of inflammation in the surrounding tissue [49]. MT staining was used to stain for collagen, which could be used to confirm if the newly regenerated tissues were bone tissues. In addition, VK staining was used to stain for calcium, which was also used to confirm the presence of bone tissue. As seen for G5.0, the area covered by the HE stain was noted to have intense MT and VK staining. Thus, it could be deduced that the gelatin-coated group was able to improve the function and bone regeneration of MgCS scaffolds, making it even more feasible for future bone tissue regeneration studies. With the use of the G5.0/FGF-2 scaffolds, new bone tissue grew from the margin around the defects, as affirmed by MT and VK staining. During the analysis of the newly formed bone tissue, it was observed that the G5.0/FGF-2 scaffold influenced significantly more bone regeneration. These results demonstrated that the appearance of the FGF-2 loaded in the gelatin-coated layer supported the bone-like environment, and the composite scaffolds provided more bone regeneration than ceramic scaffolds [50]. These results indicated that the formation of vascular lumens occurred in the G5.0/FGF-2 scaffolds, meaning that high levels of osteogenic behavior may possibly occur based on the in vivo effectiveness of FGF-2 in regulating the osteogenesis differentiation and angiogenesis activity of stem cells [51]. 

Surface coating is a simple way of modification to allow for homogenous coating of bioactive molecules such as FGF-2 in our study. The interaction between bioactive molecules and synthetic materials are more often weak, thus leading to the uneven coating of bioactive materials on surfaces of materials. Ma et al. were one of the first few who attempted to coat gelatin and bioactive molecules onto surfaces of synthetic materials [52]. It was reported in their study that immersion in gelatin allowed for penetration of gelatin into pores and the cross-linking of gelatin with glutaraldehyde further strengthened the gelatin by preventing it from falling off in the first 14 days. Similarly, to our study, their gelatin-coated scaffolds exhibited enhanced mechanical strength and hydrophilicity. In addition, their scaffold exhibited a sustained release of recombinant human bone morphogenetic protein-2 which further enhanced cellular attachment, proliferation, differentiation, and osteogenesis. Together with our results, these results demonstrate that gelatin-coated scaffolds not only can serve as functional bone substitutes that enhance the bioactivity and osteoconductivity of scaffolds, but they can also serve as loading cargoes of therapeutic agents to achieve sustained release of factors from the multi-function scaffold and consequently facilitate in vivo regeneration of bone defects. 

## 4. Conclusions

Biofabrication is a beneficial manufacturing process that creates cell-laden 3D scaffolds that further replicate the complex compositional and architectural structure of tissues. In summary, this study provides evidence of the successful construction of a gelatin-coated 3D-printed MgCS scaffold. SEM images demonstrated that the gelatin was uniformly coated on the surface of the MgCS scaffold and produced rough surfaces that influenced the cellular behavior. Among the samples, the scaffold coated with 5% gelatin (G5.0) showed good results in terms of both mechanical strength and degradation processes, which suggests a preferable physicochemical microenvironment for WJMSC adhesion and differentiation. Enhanced bone regeneration after implantation of the gelatin-coated MgCS scaffold was also observed in the µCT and histology analyses in the in vivo study. The gelatin-coated MgCS scaffold with FGF-2 presented a trend of better bone healing compared with the scaffold without the growth factor. Therefore, a gelatin-coated MgCS scaffold with FGF-2 may be a safe and feasible substitute to use as a bone void implant in bone defects. Finally, the FGF-2 in a 5% gelatin-coated layer on an MgCS scaffold demonstrated excellent bone regeneration behaviors in vivo, underscoring its potential as a novel bio-scaffold also for in situ printing. In future studies, we will focus on developing the print resolution to suit the patient-specific anatomy to make it possible to stimulate new bone regeneration by printed and fixed into the bone defect using the developed scaffold with the possibility of supplying better potential for bone tissue regeneration applications.

## Data Availability

Data are available in a publicly accessible repository.
